# Facile synthesis of ZnO/CuO/rGO nanohybrid for nonenzymatic electrochemical glucose sensor

**DOI:** 10.1039/d5ra01086a

**Published:** 2025-07-08

**Authors:** Kifle Alemu Woderasha, Getabalew Shifera Weldegrum, Shimeles Addisu Kitte, Nigus Maregu Demewoz, Beshir A. Hussein, Aknachew Mebreku Demeku, Teklu Wosenyeleh Mamo

**Affiliations:** a Department of Chemistry, Mattu University P.O.Box 318 Mettu Ethiopia getabalewsos@gmail.com; b Department of Chemistry, Jimma University P.O.Box 378 Jimma Ethiopia; c Department of Physics, Wollo University Dessie Ethiopia; d Department of Chemistry, Mekdela Amba University P.O.Box 32 Ethiopia; e Department of Materials Science and Engineering, National Taiwan University of Science and Technology Taipei 106335 Taiwan; f Department of physics, DebreBerhan University P.O. Box: 445 DebreBerhan Ethiopia; g Department of Nursing, Mettu University P.O.Box 318 Mettu Ethiopia

## Abstract

In this study, a ZnO/CuO/rGO nanohybrid was synthesized using a hydrothermal technique. The resulting nanohybrid was characterized through various methods, including UV-visible spectroscopy, XRD, and FE-SEM. The electrochemical characteristics of the sensor were examined using CV and amperometric techniques. To create the sensing electrode, the synthesized nanohybrid was applied to a glassy carbon electrode (GCE) *via* the drop-coating process. The ZnO/CuO/rGO-modified GCE demonstrated excellent electrocatalytic characteristics for glucose oxidation in an alkaline environment. Under optimal conditions, the electrochemical glucose sensor modified with ZnO/CuO/rGO/GCE exhibited a broad linear range (2–10 mM), impressive sensitivity (5660 μA mM^−1^ cm^−2^), a low detection limit (0.54 μM), and a rapid response time (3 s) for glucose detection. Moreover, the developed method displayed good repeatability (RSD = 3.8%) and stability, demonstrating its reliability.

## Abstract 2

In this study, a ZnO/CuO/rGO nanohybrid was synthesized using a hydrothermal technique.

## Graphical abstract

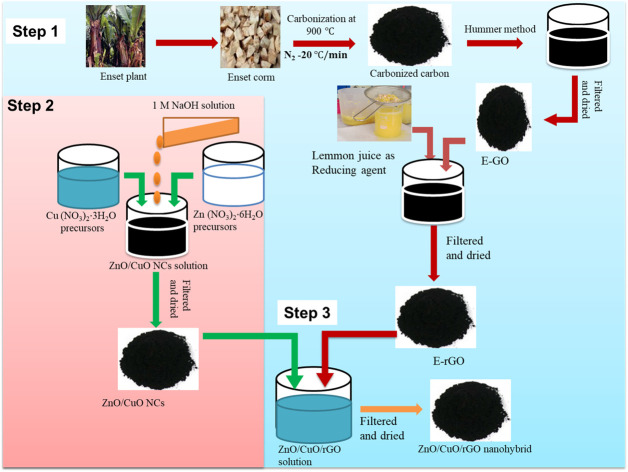

## 1. Introduction

Diabetes is a chronic disease caused by abnormal blood glucose levels, which can be life threatening if not properly managed.^1^ Glucose is vital for human health, with normal fasting blood glucose levels typically ranging from 3.9–6.1 mM (79.2–110 mg dL^−1^).^2^ Monitoring and controlling glucose levels are crucial for early diagnosis and have received increasing attention across clinical, biological, and chemical diagnostics, as well as in the textiles, wastewater treatment, environmental monitoring, and food industries,^3^ As a result, high precision glucose detection methods based on colorimetry, surface enhanced Raman scattering, fluorescence, and electrochemical techniques have been extensively explored in recent years.^4–6^ However, many of these methods are limited by long processing times, high costs, the requirement for specialized training, or insufficient sensitivity and selectivity.^7^ Among the available approaches, electrochemical glucose sensors have emerged as highly attractive alternatives due to their rapid response times, simple instrumentation, high selectivity, cost effectiveness, and potential for miniaturization and point of care applications.^8,9^

These sensors can be broadly classified into enzymatic and non-enzymatic detection strategies. Enzymatic sensors based on glucose oxidase have long been valued for their excellent selectivity, sensitivity, and biocompatibility, but they also present significant limitations, including high cost, short operational lifetime, limited electrode kinetics, poor reproducibility, and susceptibility to environmental factors such as humidity, pH, temperature, and toxic chemicals.^10,11^ These constraints have motivated a shift toward the direct detection of glucose using nonenzymatic electrochemical sensors.^12,13^

Non enzymatic glucose sensors offer notable advantages over enzyme based alternatives, including improved cost effectiveness, reproducibility, stability, and resistance to thermal and chemical variations.^14,15^ In such sensors, glucose is oxidized directly on the electrode surface, which is typically modified with nanomaterials. However, challenges remain, including high working potentials, unpredictable redox behavior, slow electron transfer kinetics, surface poisoning, and relatively low detection sensitivity.^16^

To overcome these limitations, significant efforts have been dedicated to designing advanced nanomaterials with high catalytic activity, enhanced conductivity, and excellent physicochemical stability. The favorable characteristics of these materials including their large surface area, well defined porosity, low charge transfer resistance, and structural adaptability make them highly promising candidates for electrode materials.^17,18^

A range of nanostructured electro catalysts for glucose oxidation have been extensively studied, including conductive polymer,^15^ transition metals,^19^ noble metals,^20^ bimetallic systems,^21^ and their nanocomposites (NCs) based on carbon nanomaterials.^22^ These materials are widely used in enzyme free electrochemical glucose sensors, with transition metal and metal oxide based electrodes gaining particular attention due to their affordability, high conductivity, and robust catalytic activity.^21^ Among these, heterostructured composites of ZnO and CuO stand out for their remarkable electrochemical performance, low cost, long term stability, biocompatibility, and ability to form p–n junctions that expand the electron depletion region, thereby boosting charge transfer efficiency and overall sensor performance.^23^ Moreover, NCs based on nanocarbon materials, such as CuO NPs/rGO,^24^ Cu_2_O NPs/rGO,^25^ CuO NPs/nitrogen doped graphene (NGP),^26^ and CuO/graphene oxide (GO),^27^ have demonstrated excellent electrocatalytic performance for glucose detection.

In recent years, the use of conductive materials, including carbon nanotubes (CNTs) and graphene derivatives, has gained significant attention for enhancing the electrocatalytic efficiency of ZnO/CuO based sensors.^17^ Incorporation of nanocarbon supports can dramatically increase the surface area, enable higher catalyst loading, and thereby boost the sensor's reactivity and sensitivity toward glucose oxidation.^28^

Graphene, a two dimensional material composed of a single layer of sp^2^-hybridized carbon atoms, has attracted widespread interest due to its unique physical and chemical properties, such as a large surface area, ease of functionalization, high electrical conductivity, and strong biocompatibility.^29,30^ These characteristics make graphene an ideal candidate for biological detection and analysis.^31,32^ In the context of non-enzymatic sensors, graphene improves the stability and reliability of glucose detection. For example, Shang *et al.*,^33^ developed multilayer graphene nanoflake films on silicon *via* microwave assisted plasma chemical vapor deposition for the detection of uric acid, ascorbic acid, and dopamine. Similarly, Kim *et al.*,^34^ demonstrated the successful adsorption of graphene onto a glassy carbon electrode, achieving distinct electrochemical detection of dopamine, ascorbic acid, and uric acid, while Yu-Wei Hsu *et al.*,^35^ combined ZnO nanorods with graphene for similar applications.

Although commercially available graphene derivatives are highly effective, their high cost has limited their widespread use in sensor fabrication. To overcome this challenge, researchers have increasingly focused on synthesizing nanostructured materials from bio waste sources, including activated carbon and graphene derivatives.^36^ Techniques such as mechanical exfoliation, chemical synthesis, and chemical vapor deposition (CVD) have been used for this purpose.^36,37^ However, while CVD is suitable for large scale production, it is not cost effective. Meanwhile, traditional chemical synthesis often involves toxic reducing agents such as hydrazine hydrate and sodium borohydride. To address these limitations, cost effective and ecofriendly synthesis methods have emerged as promising alternatives for industrial,^38,39^ environmental,^40^ and biological^41^ applications. For example, Medha Gijare *et al.*,^42^ demonstrated the synthesis of reduced graphene oxide using a low cost plant extract (lemon juice) as a reducing and stabilizing agent for use in non-enzymatic glucose sensors. These advances are especially valuable for applications in environmental monitoring, clinical diagnostics, and pharmaceutical investigations. Over the past decade, a range of techniques including *in situ* chemical synthesis, hydrothermal methods, microwave heating, and electro deposition have been employed for the commercial production of graphene derivatives combined with metal or metal oxide NPs and polymer based NCs for non-enzymatic glucose detection.^43–46^

In this work, we present a bio waste derived rGO material and a green synthesis approach as an ecofriendly and cost effective alternative to commercially available graphene and traditional chemical methods for the fabrication of a non-enzymatic glucose sensor. By drop casting a ZnO/CuO/rGO nanohybrid onto a glassy carbon electrode, we developed a highly sensitive and sustainable glucose sensing platform. Compared with previously reported analogs, the proposed sensor demonstrated a wider linear range suitable for blood glucose monitoring, a lower detection limit, and higher sensitivity. Its exceptional electrocatalytic activity towards glucose is attributed to the superior electrical conductivity of rGO, which promotes rapid electron transfer between the electrode surface and the analyte. The optical, structural, and morphological characteristics of the synthesized nanohybrid were thoroughly examined, and its performance was evaluated using cyclic voltammetry (CV) and chronoamperometry (CA).

## 2. Experimental

### 2.1. Materials and chemicals

The materials used included lemon fruit (sourced from the local market in Mattu, Ethiopia) and Enseteventricosum corm waste (obtained from Kaffa Zone, Southwest Ethiopia). Blood samples were collected in sterile glass bottle (a gift from a diabetic male patient (Haile Woderasha Abi), Kaffa Zone, and Southwest Ethiopia. The chemicals used included glucose (C_6_H_12_O_6_), sodium hydroxide (NaOH), sodium carbonate (Na_2_CO_3_), maltose (C_12_H_22_O_11_), and sucrose (C_12_H_22_O_11_) from BDH Chemicals; zinc nitrate hexahydrate (Zn(NO_3_)_2_·6H_2_O), ascorbic acid (C_6_H_8_O_6_), and uric acid (C_5_H_4_N_4_O_3_) from Blulux Laboratories; hydrogen peroxide (H_2_O_2_, 30%), potassium permanganate (KMnO_4_, 99%), sulfuric acid (H_2_SO_4_, 99.99%), hydrochloric acid (HCl, 30%), glucose, and dopamine from Qualigens Fine Chemicals, India. Alumina powder with particle sizes of 1 μm, 0.3 μm, and 0.05 μm was procured from Sigma Aldrich and Merck, Germany. The phosphate buffer solution (PBS) was prepared in the laboratory using established protocols. All chemicals were of analytical grade and used as received, without further purification.

### 2.2. Synthesis of ensetventricosum corm-derived graphene oxide (GO)

The synthesis of GO from Enset ventricosum corm was performed based on earlier reports, with some modifications.^47–50^ Initially, the Enset ventricosum corm were isolated from Enset ventricosum, rinsed with purified water, and dried in an oven at 105 °C for 24 h. The dried corm were then finely ground and calcined at 900 °C for 3 h under a nitrogen atmosphere to obtain Enset ventricosum derived carbon for GO synthesis. In a typical reaction, 5 g of the Enset ventricosum corm powder and 6 g of NaNO_3_ were mixed with 150 mL of concentrated H_2_SO_4_ and stirred continuously for 60 minutes. Then, 15 g of KMnO_4_ were added gradually to the mixture in an ice bath (0–5 °C) and stirred for 3 h. The resulting mixture was kept at room temperature and stirred for an additional 24 h until the color changed from black to purplish brown, indicating the completion of the oxidation process. After this, 150 mL of distilled water was added, and the mixture was heated to 90 °C for 20 minutes to obtain a dark brown solution. Upon cooling to room temperature, another 200 mL of water was added, and the reaction was stopped by adding 20 mL of H_2_O_2_ to remove excess KMnO_4_. The resulting GO was washed with distilled water and ethanol until neutral pH (pH 7) and dried in an oven at 40 °C for 36 h.

### 2.3. Reduction of GO with aqueous lemon peel extract

The synthesis of rGO from Enset ventricosum corm was performed with modifications based on prior research.^42^ Fresh lemons were purchased from a local market, washed with purified water, and peeled. The peels were blended using a household mixer and soaked overnight in distilled water. The mixture was then heated to 50 °C, stirred for 30 minutes, and filtered, and the resulting solution was stored at 4 °C for future use. Meanwhile, 50 mg of GO was dispersed in 0.1 mg mL^−1^ of purified water and sonicated for 45 minutes. Then, 10 mL of the lemon peel extract was added to the GO solution, and the mixture was refluxed at 90 °C for 6 h, yielding a brownish black suspension. The supernatant was collected by centrifugation at 4000 rpm and dried at 100 °C in a vacuum oven.

### 2.4. Synthesis of ZnO nanoparticles

ZnO NPs were synthesized *via* the precipitation method as described in earlier reports.^51^ In a typical procedure, 3.7138 g of Zn (NO_3_)_2_·6H_2_O was dissolved in 200 mL of distilled water to prepare a 0.0625 M Zn (NO_3_)_2_ solution. Similarly, 2.248 g of (NH_4_)_2_CO_3_·3H_2_O was dissolved in 240 mL of purified water to obtain a 0.0625 M (NH_4_)_2_CO_3_ solution, which was stirred for 15 minutes at room temperature. The (NH_4_)_2_CO_3_ solution was then added dropwise to the Zn (NO_3_)_2_ solution under continuous stirring for 1 hour. The resulting zinc carbonate precipitate was filtered under vacuum and washed thoroughly with distilled water and ethanol to remove any NH_4_NO_3_ byproduct, unreacted (NH_4_)_2_CO_3_, and residual solvents. The solid was dried at 80 °C overnight to remove adsorbed moisture and other volatiles, and then calcined at 500 °C for 2 h to decompose the ZnCO_3_ into ZnO NPs. The resulting ZnO NPs was stored in a sealed plastic container to prevent moisture and air exposure.

### 2.5. Synthesis of ZnO/CuO nanocomposites

ZnO/CuO NCs with a 1 : 1 molar ratio were synthesized using Cu (NO_3_)_2_·3H_2_O and Zn (NO_3_)_2_·6H_2_O as precursors. The salts were dissolved in 100 mL of purified water to obtain a 0.2 M mixed metal nitrate solution. A 1 M NaOH solution was then added dropwise under vigorous stirring until the pH reached 14. The resulting alkaline mixture was stirred at 80 °C until a black precipitate formed. The precipitate was collected, washed 2–3 times with distilled water and absolute ethanol, and dried overnight in an oven at 80 °C. Finally, the dried precipitate was calcined at 450 °C for 3 h to obtain the ZnO/CuO NCs.^52^

### 2.6. Synthesis of ZnO/CuO/rGO nanohybrid

**Fig. 1:**
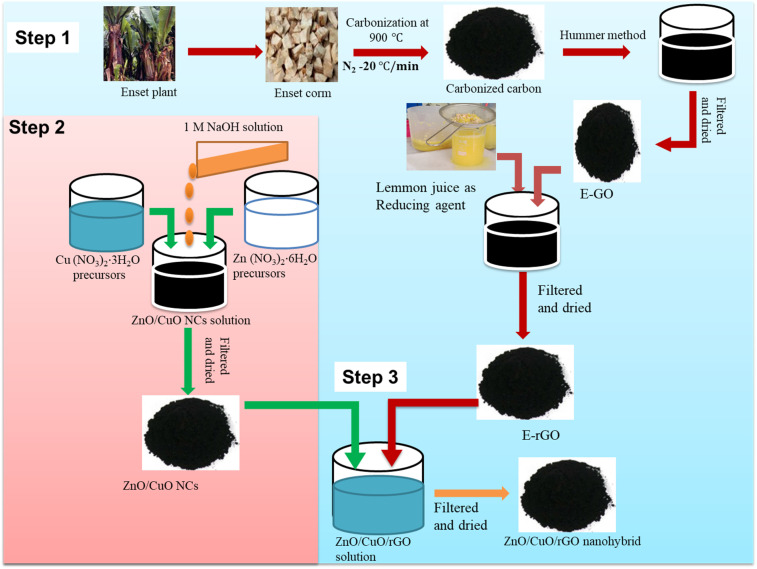
Schematic diagram for the synthesis of ZnO/CuO/rGO nanohybrid.

The ZnO/CuO/rGO nanohybrid was prepared using a modified method based on the procedures outlined in ref. 44 and 53. In a typical experiment, 5 mg of as synthesized rGO was dispersed in 60 mL of ethanol and sonicated for 2 h. Meanwhile, 6.3 g of the synthesized ZnO/CuO NCs was dispersed in 100 mL of distilled water and stirred at 55 °C for 2 h to obtain a uniform suspension. The ZnO/CuO NCs suspension was then added dropwise to the rGO solution under a nitrogen atmosphere at room temperature with continuous stirring. The resulting mixture was collected by centrifugation, washed thoroughly with deionized water and ethanol to remove any impurities, and dried at 80 °C overnight to obtain the ZnO/CuO/rGO nanohybrid. Fig. 1 illustrates the synthesis process of the ZnO/CuO/rGO nanohybrid.

### 2.7. Preparation of modified carbon electrode

Prior to surface modification, the glassy carbon electrodes (GCEs) were sequentially polished with alumina slurries of 1 μm, 0.3 μm, and 0.05 μm particle sizes to obtain a clean, smooth surface. After each polishing step, the electrodes were rinsed thoroughly with distilled water. The polished GCEs were then sonicated for 5 minutes in a 1 : 1 mixture of nitric acid, ethanol, and purified water, and dried at room temperature. The as-prepared nanoparticles, nanocomposites, and nanohybrid (5 mg) were dispersed in a mixture of 3 mL deionized water and 2 mL ethanol by ultrasonic agitation. An 8 μL aliquot of the resulting ZnO/CuO/rGO suspension was drop cast onto the GCE surface and dried in an air oven at 35 °C until a uniform film was formed. Similarly, control electrodes (ZnO/GCE, CuO/GCE, and ZnO/CuO/GCE) were prepared following the same experimental procedure.^52^

### 2.8. Materials characterization

The synthesized materials (ZnO, ZnO/CuO, and ZnO/CuO/rGO) were characterized using a range of analytical techniques. X-ray diffraction (XRD) was performed with a Bruker D2 Phaser (Germany) using Cu Kα radiation (*λ* = 1.5405 Å) at 30 kV and 10 mA, with a scan range of 10–80°. Scanning electron microscopy (SEM) and energy dispersive X-ray spectroscopy (EDX) were conducted using a JEOL JSE-6700F (Japan) at an accelerating voltage of 15 kV. UV-visible spectroscopy was used to investigate the optical properties of the samples, while Fourier transform infrared spectroscopy (FTIR) was performed on a Shimadzu IRTracer-100 (Japan) over the range of 4000–400 cm^−1^.

Cyclic voltammetry (CV) and amperometric measurements were conducted using a BAS100B Electrochemical Bioanalyzer with Windows™ software. The three electrode configuration consisted of a modified glassy carbon working electrode, a platinum wire counter electrode, and a leak free Ag/AgCl reference electrode. All electrochemical measurements were performed in 0.1 M NaOH, with or without 2 mM glucose, within a potential window of −0.2 to 0.1 V at a scan rate of 50 mV s^−1^. For amperometric glucose detection, glucose was added incrementally under magnetic stirring, and the current response was recorded at an applied potential of 0.55 V in a magnetically stirred NaOH electrolyte.

### 2.9. Electrochemical performance of the sensor

To evaluate the reproducibility, repeatability, and stability of the ZnO/CuO/rGO nanohybrid modified electrodes, multiple electrodes were prepared and tested under identical conditions. To assess reproducibility, glucose detection was performed five times using a single electrode, with the surface thoroughly rinsed with deionized water after each measurement, and the standard deviation of the electrochemical current response was calculated. To confirm repeatability, five independently prepared electrodes were tested for glucose detection, yielding consistent results across all samples, with the average current and standard deviation recorded. Long term stability was evaluated by monitoring the current response of the ZnO/CuO/rGO modified electrodes to glucose over a period of 10 days. Measurements were taken every 2 days, with electrodes stored under ambient conditions between measurements. The results demonstrated that the sensor retained a stable and reproducible current response throughout the testing period.

### 2.10. Measurement of glucose in real samples (human blood serum)

The performance of the proposed sensor was evaluated by measuring glucose levels in human blood samples. The glucose concentration was first determined spectrophotometrically from three replicate measurements. Prior to electrochemical analysis, the blood samples were centrifuged at 3000 rpm for 15 minutes to remove precipitated proteins and other particulate matter. A 0.1 mL aliquot of the resulting serum was then diluted with 10 mL of 0.1 M NaOH solution. The electrochemical measurements were performed using the ZnO/CuO/rGO modified GCE at the optimal applied potential. The accuracy of the method was further confirmed by recovery studies. All experiments were conducted at room temperature, and the recovery percentages were calculated using the following formula:1
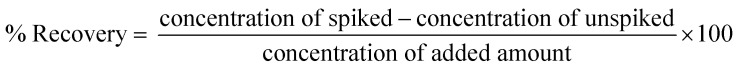


## 3. Results and discussion

### 3.1. XRD analysis

**Fig. 2:**
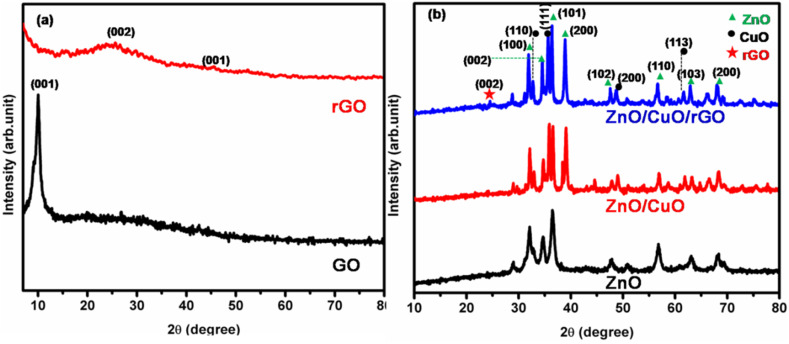
(a) XRD pattern of GO, and rGO, (b) XRD patterns of ZnONPs, ZnO/CuO NCs, and ZnO/CuO/rGOnanohybrid.

XRD is a widely used, non-destructive technique for determining lattice spacing, crystallite sizes, and preferred orientations of materials. To confirm the successful synthesis of GO, rGO, ZnO/CuO, and ZnO/CuO/rGO, XRD analyses were performed, as shown in Fig. 2. The XRD pattern of GO exhibited a sharp, intense (001) reflection at 2*θ* = 10° (Fig. 2(a)), indicating effective oxidation and the introduction of oxygen containing functional groups into the graphitic structure.^54^ The successful conversion of GO to rGO using lemon peel aqueous extract as a reducing and stabilizing agent was confirmed by a broad XRD peak at 2*θ* = 24.24°, corresponding to the (002) lattice reflection, indicating the formation of rGO.^9^ Furthermore, the (002) plane of GO disappears in rGO, suggesting *in situ* reduction of GO to rGO.^55^ A similar broad peak was observed in the angle range between 15–30 during the synthesis of rGO using heated coconut shells.^56^ In the XRD pattern shown in Fig. 2(b), the ZnO diffraction peaks appeared at approximately 2*θ* = 29.07°, 31.80°, 34.89°, 36.48°, 47.57°, 57.16°, 63.07°, and 68.56°, corresponding to the (100), (002), (101), (102), (110), (103), (200), and (112) lattice planes, respectively. These peaks are characteristic of the hexagonal wurtzite structure of ZnO (JCPDS No. 36-1451), in agreement with previous studies.^57^ In the XRD pattern of the ZnO/CuO NCs, six characteristic peaks of CuO appeared at approximately 2*θ* = 32.66°, 36.80°, 38.38°, 48.80°, 62.35°, and 68.45°, corresponding to the (002), (111), (202), (121), (222), and (212) lattice planes (JCPDS No. 48-1548), along with peaks attributable to the hexagonal wurtzite structure of ZnO. In the XRD pattern of the ZnO/CuO/rGO nanohybrid, a broad, low intensity peak at approximately 2*θ* = 25.75° confirmed the presence of rGO. No additional peaks were observed, indicating the successful synthesis of the ZnO/CuO/rGO nanohybrid with a well crystallized structure and high phase purity.

The crystal sizes, *d*-spacing of distinct materials, and distinct shifts for ZnO NPs, ZnO/CuO NCs, and ZnO/CuO/rGO nanohybrid materials are listed in Table 1.2
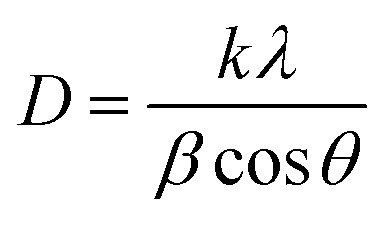
3
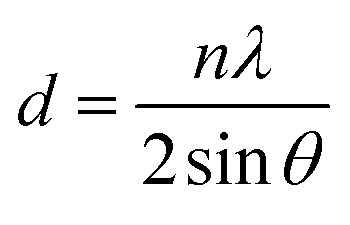
where *D* is the average crystallite size, *d* is the plane spacing, *θ* is the Bragg angle, *λ* is the X-ray wavelength, and *β* is the full width at half maximum (FWHM). The crystallite sizes of ZnO, ZnO/CuO, and ZnO/CuO/rGO nanohybrid were 20.25, and 16.81 respectively. The average particle size of ZnO/CuO/rGO was smaller than that of ZnO/CuO. The ternary ZnO/CuO/rGO nanohybrid showed the highest surface area because of the synergistic effect of the ZnO, CuO, and rGO components in the composite system, which reduces the particle size. A decrease in particle size of NCs is primarily shown by the widening of diffraction peaks due to reduced coherent crystal size. Confirms the slight shift in peak positions, showing changes in lattice parameters, while variations in relative peak intensities can reflect changes in crystal orientation distributions.

**Table 1:** Crystal parameters of GO, rGO, ZnO, CuO/ZnO and ZnO/CuO/rGO

Sample name	2*θ*	FWHM	Crystal size nm	Average crystal size	*d*-spacing	*hkl*
GO	9.85	1.009	1.000	7.90	0.066	011
rGO	24.24	1.262	1.062	13.29	0.162	002
	45.89	1.065	0.455		0.300	001
ZnO	31.91	1.404	5.883	20.25	0.212	100
	34.67	0.518	16.057		0.230	002
	36.40	0.406	20.581		0.241	101
	56.80	0.235	38.493		0.366	110
ZnO/CuO	32.15	0.532	15.541	16.81	0.213	100
	36.00	0.599	13.950		0.238	101
	38.09	0.364	23.119		0.251	200
	57.04	0.620	14.646		0.373	110
ZnO/CuO/rGO	32.93	0.327	25.311	16.18	0.218	100
	36.93	1.543	5.427		0.244	101
	38.90	0.344	24.466		0.257	200
	56.72	0.948	9.526		0.366	110

On the other hand, variations in the full width at half maximum (FWHM) of XRD peaks in nanohybrid shows important structural features. An increase in FWHM suggests a reduction in crystallite size, as smaller crystals yield broader peaks. High FWHM values can also signify the presence of internal strain or structural defects within the material, as well as reflect alterations in crystalline or phase composition. In the provided XRD data in tabulated in Table 1, higher FWHM values for ZnO/CuO and ZnO/CuO/rGO samples compared to pure ZnO emphasize smaller crystallite sizes associated with the addition of CuO and rGO. This increase in FWHM may also show heightened internal strain and changes in crystallography resulting from interactions among the components. Moreover, the observed variations may imply that the introduction of new phases disrupts the ordered structure of the ZnO crystals. Overall, these findings highlight how integrating CuO and rGO not only contributes to smaller crystallite sizes but also enhances strain and disorder within the ZnO framework, potentially impacting properties like photo catalytic activity, conductivity, and mechanical strength.

### 3.2. SEM–EDS analysis

**Fig. 3:**
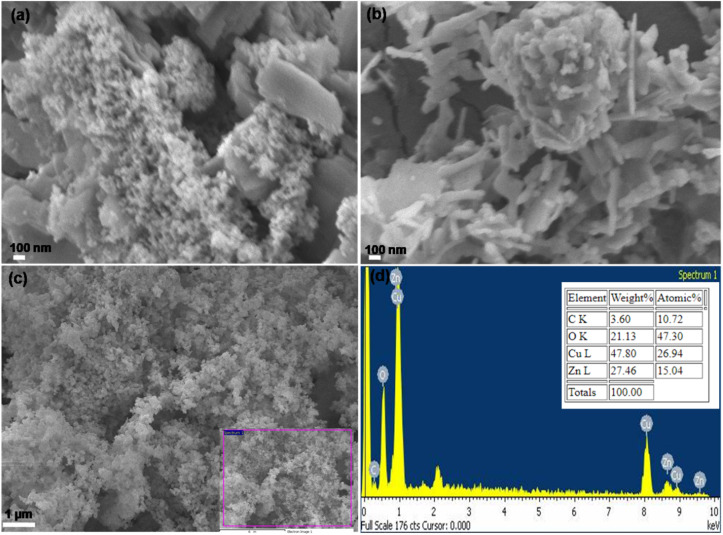
SEM images ZnO NPs (a), ZnO/CuO NCs (b), CuO/ZnO/rGO nanohybrid (c) and ED's spectra of the CuO/ZnO/rGO nanohybrid (d).

The as-synthesized ZnO NPs, ZnO/CuO NCs, and ZnO/CuO/rGO nanohybrid' surface morphology were studied using SEM at various magnifications, as presented in Fig. 3. Fig. 3(a) and (b) are the SEM images of ZnO and CuO/ZnO samples. When minor ZnO NPs are present, the surface morphology of the CuO nanoplatelet changes, suggesting that the ZnO NPs are uniformly dispersed on the CuO nanoplatelet surface. The existence of ZnO on the CuO nanostructure forms a CuO/ZnO heterojunction with a complex shape and clearly promotes dynamic adsorption of the reacting molecules. On the other hand, the SEM micrograph of the ZnO/CuO/rGO nanohybrid in Fig. 3(c) shows that the ZnO NPs and CuO plates are uniformly distributed and interconnected on the rGO sheet nanostructure.

Furthermore, the SEM images reveal the distinct morphological characteristics and structural evolution of the synthesized materials. The first ZnO NPs, shown in Fig. 3(a), display aggregated clusters with irregular shapes and sizes, exhibiting a rough, granular surface texture with significant particle agglomeration. Upon incorporation of CuO to form CuO/ZnO NCs, as depicted in images b, a remarkable transformation in morphology is observed. The composite structure develops into a more sophisticated flower-like or hierarchical arrangement, characterized by distinctive plate-like or leaf-like projections extending from the central structures. This modification proves enhanced dispersion suggesting successful integration of the two metal oxide components.

The final stage of the composite synthesis, involving the addition of rGO to form CuO/ZnO/rGO (Fig. 3(c), exhibits the most complex morphological features. The resulting structure reveals an interconnected network architecture where the metal oxide components appear to be effectively anchored onto the rGO sheets. This arrangement creates a layered structure with visible porosity, showing potential enhancement in surface area compared to its precursor materials. The SEM analysis confirms the successful synthesis of the hybrid material, showing good integration between all components. The progressive evolution of morphology throughout the synthesis stages suggests that the final composite structure may offer improved performance characteristics for various applications, attributed to its hierarchical organization and enhanced structural features. The EDS spectra of the ZnO/CuO/rGO sample, which substantiates the elemental composition and purity of the as prepared composite materials. The EDS analysis exhibits that the constituents of ZnO/CuO/rGO included mainly Zn, Cu, O, and C. This shows those ZnO/CuO/rGO nanohybrid was successfully produced (Fig. 3(d)).

### 3.3. UV-vis and FTIR spectra analysis

**Fig. 4:**
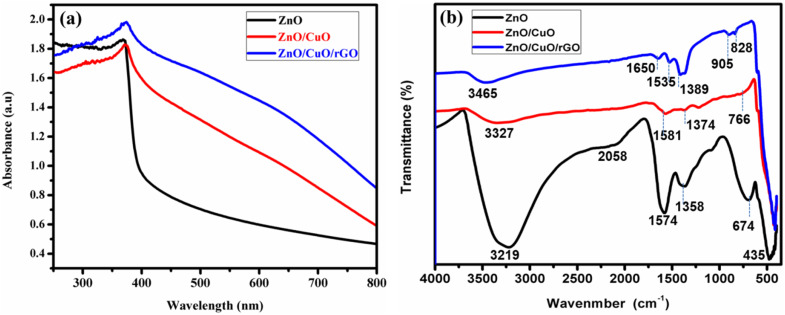
UV-vis (a) and FTIR spectrum (b) of ZnO NPs, ZnO/CuO NCs, and ZnO/CuO/rGO nanohybrid.

Fig. 4 presents the absorption spectra in the UV-visible range for the ZnO NPs, ZnO/CuO NCs, and ZnO/CuO/rGO, with measurements taken at ambient temperature. The UV-vis spectrum (Fig. 4(a)) reveals a distinctive sharp peak centered at 368 nm, which corresponds to a 3.1 eV band gap, typical of ZnO nanostructures. This band gap energy aligns with established values for ZnO.^45^ The absorption peak at 372 nm indicates a ZnO/CuO NCs with a band gap value of 2.75 eV; the band gap of ZnO NPs declined from 3.1 eV to 2.75 eV for the ZnO/CuO NCs. The 374 nm absorption peak shows the ZnO/CuO/rGO nanohybrid materials, with the band gap decreasing to 2.68 eV; the ZnO/CuO NCs and the ZnO/CuO/rGO nanohybrid show enhanced absorbance in the visible light region.^46^ The spectra of ZnO/CuO/rGO nanohybrid showed a wider background in the visible region, due to the presence of rGO; the absorbance of the ZnO/CuO/rGO nanohybrid was seen to increase compared to ZnO NPs and ZnO/CuO NCs. The experimental data show that the band gap of ZnO decreased when combined with ZnO, CuO, and rGO. The optical band gap energy of each composite was calculated by employing the Tauc model in the high absorption zone, given by the following relationship.

4 (*αhυ*)^*n*^ = *K*(*hυ* − *E*_g_)

where *α* is the absorption coefficient, *hν* is the photon energy, *K* is a proportionality constant that differ with the material, *n* is the exponent or optical transition.

FTIR spectroscopy was performed in the range of 400–4000 cm^−1^ to investigate the chemical bonding and functional groups present in the materials. The FTIR spectra of ZnO NPs, ZnO/CuO NCs, and ZnO/CuO/rGO nanohybrid (Fig. 4(b)) reveal distinct vibrational modes, confirming the successful synthesis and integration of each component. In the ZnO spectrum, the broad, intense band at approximately 3220 cm^−1^ is attributed to the O–H stretching vibrations of surface-adsorbed water and hydroxyl groups, indicating the highly polar nature of the NPs.^58^ The band at ∼1574 cm^−1^ is associated with the C=O stretching vibrations of carboxylate groups adsorbed on the surface.^59^ while the signal at ∼1358 cm^−1^ is ascribed to the asymmetric stretching of nitrate species (NO_3_^−^) from the precursor.^60,61^ The sharp peaks at ∼435, 674, and 766 cm^−1^ correspond to the characteristic metal–oxygen lattice vibrations of Zn–O, confirming the presence of the ZnO phase.^61–63^

In the ZnO/CuO NCs, the dominant O–H stretching vibration and other spectra peaks of ZnO are slightly modified due to the interaction between CuO and ZnO, resulting in a positive shift and change in intensity. New vibrational modes, particularly near 766 cm^−1^, arise from Cu–O stretching, indicating successful incorporation of CuO within the ZnO lattice.^64^

The broad band at ∼3465 cm^−1^ in the ZnO/CuO/rGO composite is attributed to the O–H stretching vibrations of adsorbed water and surface-bound hydroxyl groups, reflecting the hydrophilic nature and residual oxygen containing groups introduced by the rGO.^65,66^ Additional peaks observed at ∼1650 correspond to the C=C stretching vibrations of the graphitic domains of reduced graphene oxide (rGO), confirming its successful reduction and integration within the nanohybrid. These peaks are indicative of the sp^2^ hybridized carbon lattice.^66,67^ Signals between ∼1535 and 1389 cm^−1^ arise from C–O–C stretching and other oxygen containing groups associated with rGO.^68–70^ Moreover, the bands observed at approximately 905 and 825 cm^−1^ can be attributed to lattice vibrations associated with Cu–O–Zn linkages and oxygen deficient metal–oxygen modes, confirming the coupling between the metal oxides and the rGO support. Overall, the FTIR results clearly demonstrate the successful synthesis of the ZnO/CuO/rGO nanohybrid, preserving the characteristic vibrational modes of each component. The synergistic combination of the metal oxides and the rGO support is expected to enhance the material's electronic, catalytic, and surface adsorption properties, making it highly suitable for sensor and energy-related applications.

### 3.4. Electrochemical characterization

#### 3.4.1. Electro catalytic oxidation of glucose on modified electrodes

**Fig. 5:**
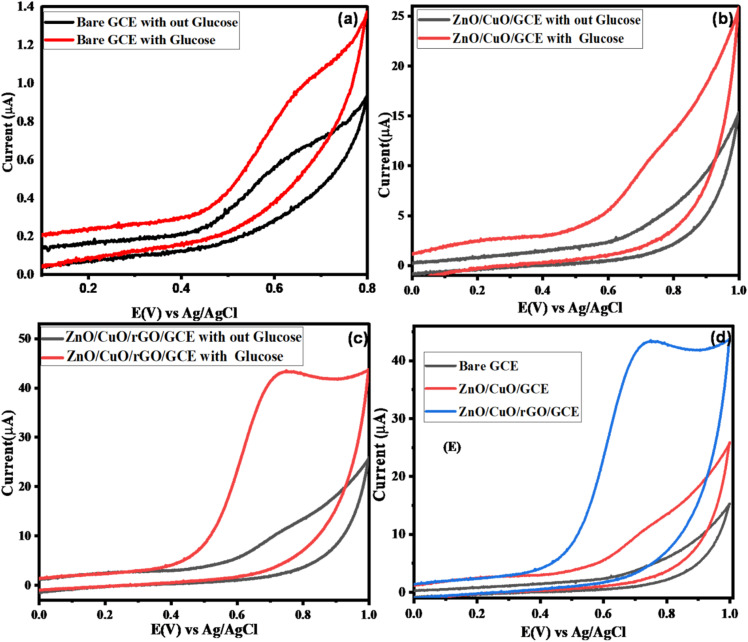
Cyclic voltammograms of different electrodes: (a) bare GCE, (b) ZnO/CuO/GCE, and (c) ZnO/CuO/rGO/GCE, in the absence and presence of 2 mM glucose, and (d) comparison of the studied electrodes in the presence of 2 mM glucose, all recorded in 0.1 M NaOH at a scan rate of 50 mV s^−1^.

The electrocatalytic activity of the bare and modified electrodes toward glucose oxidation was evaluated using cyclic voltammetry (CV) in 0.1 M NaOH, both in the absence and presence of 2 mM glucose. The measurements were performed over a potential range of −0.2 V to 0.1 V *vs.* Ag/AgCl at a scan rate of 50 mV s^−1^. Fig. 5 presents the CV profiles of the bare GCE (a) and the electrodes modified with an equal amount (0.016 g) of active material: ZnO/CuO/GCE (b) and ZnO/CuO/rGO/GCE (c), in 0.1 M NaOH, with and without 2 mM glucose.

In the absence of glucose, both the bare and modified electrodes exhibited similar CV profiles, indicating no significant background activity. However, upon the addition of glucose, the bare GCE showed no noticeable oxidation response, whereas the ZnO/CuO/GCE and ZnO/CuO/rGO/GCE demonstrated well-defined oxidation peaks at approximately +0.67 V and +0.65 V, respectively (Fig. 5b and c). The slight negative shift in over potential is attributed to the synergistic catalytic effects of rGO and the ZnO/CuO NCs, which expand the electroactive surface area and enable more efficient electron transfer between glucose and the modified electrodes.

The enhanced electrocatalytic oxidation of glucose in alkaline conditions, along with improved sensor sensitivity, can be ascribed to the combined effects of the CuO/ZnO nanocomposites and the rGO support. Notably, the ZnO/CuO/rGO/GCE exhibited a higher anodic peak current compared to both the bare GCE and the ZnO/CuO/GCE, highlighting its superior performance at the same scan rate.

Fig. 5(d) further confirms that the ZnO/CuO/rGO/GCE achieved improved electro catalytic activity due to its increased surface area, accelerated electron transport, and robust mechanical support arising from the combined properties of ZnO, CuO, and rGO. The unique electrical characteristics of ZnO enable more efficient charge transport between the redox probe and the electrode surface, making it an ideal inorganic matrix for anchoring CuO NPs. Meanwhile, rGO provides an extensive surface area for higher catalyst loading and improved conductivity. Together, these features yield a nanohybrid with a high surface area-to-volume ratio, significantly enhancing its interaction with glucose and overall sensor performance.

#### 3.4.2. Mechanism of ZnO/CuO/rGO nanohybrid glucose sensing

**Fig. 6:**
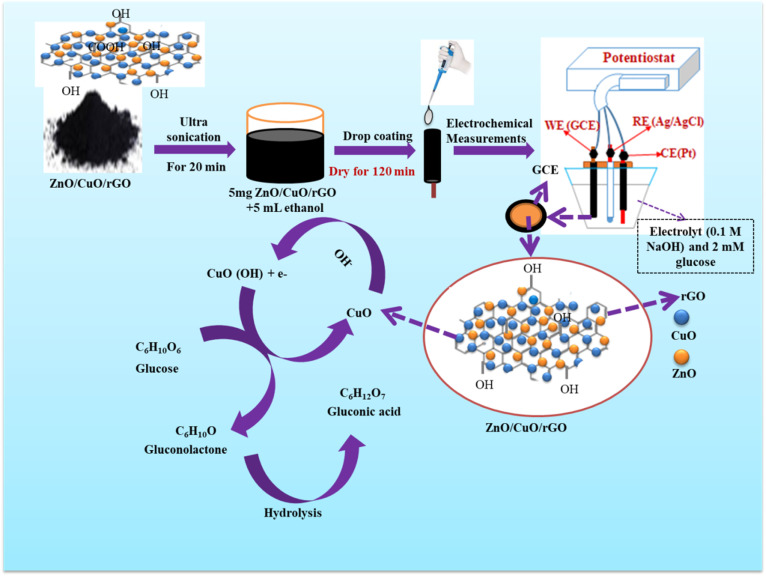
Schematic diagrams for the preparation of modified glass carbon electrode and mechanism for the glucose electro oxidation in an alkaline solution.

Fig. 6 illustrates the fabrication of the ZnO/CuO/rGO nanohybrid and its application in glucose sensing. The electrocatalytic oxidation of glucose at the ZnO/CuO/rGO modified electrode is believed to occur *via* a multi-step mechanism, involving the active CuO and Cu(OH)_2_ species present within the NCs. According to previous studies,^71^ the commonly accepted mechanism of glucose electro oxidation in the 0.1 M NaOH solution can be described using the following process.

The n-type ZnO improves the intrinsic properties of the p-type CuO and also serves as a robust mechanical support. The sensitivity and limit of detection (LOD) of the ZnO–CuO/rGO/GCE sensor are enhanced by the rapid electron transfer arising from the p–n junction between the ZnO–CuO NCs and the high surface area of rGO.

The mechanism of the CuO-based non-enzymatic glucose sensor is primarily attributed to the semiconducting characteristics of CuO nanoparticles and the adsorption of hydroxyl ions (OH^−^). An increase in the anodic potential of CuO (a p-type semiconductor) distorts the electron cloud around its oxygen atoms, facilitating the adsorption of OH^−^. The accumulated energy around the adsorbed OH^−^ promotes its affinity for glucose oxidation. In this process, CuO–OH first oxidizes glucose (C_6_H_12_O_6_) to gluconolactone (C_6_H_10_O_6_), which is subsequently hydrolyzed to gluconic acid (C_6_H_12_O_7_). This catalytic mechanism can be expressed as follows.^27,72,73^

5 CuO (s) + OH^−^ (aq) → (CuO–OH^−^) (aq) + e^−^

6 CuOOH (s) + glucose (aq) → Cu (OH)_2_ (aq) + gluconolactone (aq)

The strong oxidizing Cu(iii) species act as mediators in the process of electron transfer and nano-copper NPs substantially improve the response signal for glucose due to their strong catalytic effects.6.1
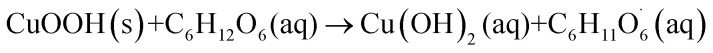


Glucose radical intermediate6.2
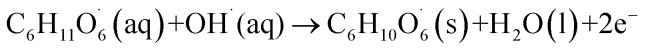


Radical intermediate gluconolactone7
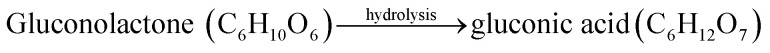


#### 3.4.3. Effect of scan rate on the electrochemical oxidation of glucose

**Fig. 7:**
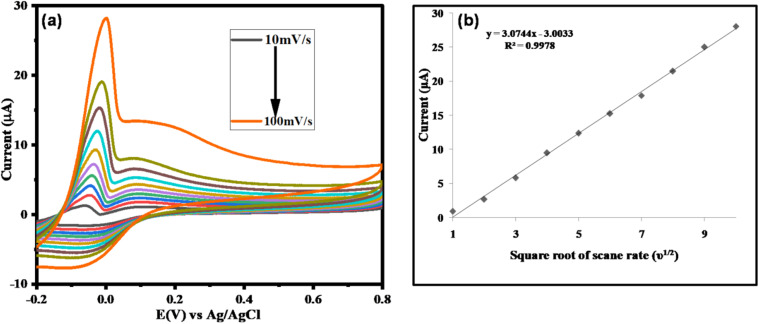
(a) Effect of scan rate (10–100 mV s^−1^) on the cyclic voltammograms of ZnO/CuO/rGO in the presence of 0.1 M NaOH and 2 mM glucose, (b) the plot of ZnO/CuO/rGO/GCE current against the square of scan rate (*ν*^1/2^).

To investigate the kinetics of glucose oxidation, the ZnO/CuO/rGO modified electrodes were examined using cyclic voltammetry (CV) at different scan rates. Fig. 7(a) shows the CV curves of the ZnO/CuO/rGO electrode at scan rates ranging from 10 to 100 mV s^−1^ in 0.1 M NaOH containing 2 mM glucose. As the scan rate increased, the anodic peak current rose linearly, and the peak potential shifted slightly toward more positive values.

**Fig. 8:**
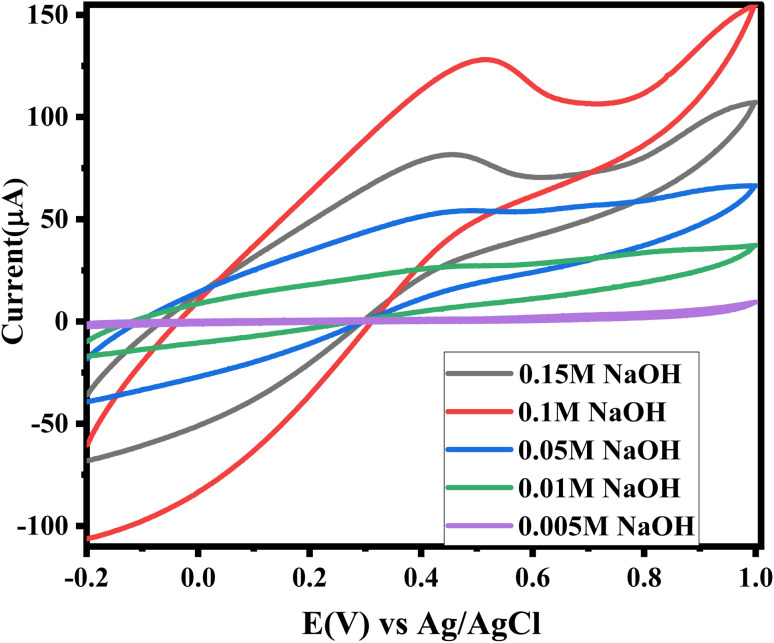
CV of ZnO/CuO/rGO/GCE *versus* at different concentration of NaOH (0.005 M up to 0.15 M) upon addition of 2 mM glucose at scan rate of 50 mV s^−1^.

This behavior is attributed to the fact that the thickness of the diffusion layer is influenced by the scan rate, with lower scan rates yielding thicker diffusion layers. When the anodic peak current (ipa) was plotted against the square root of the scan rate (*ν*^1/2^), a linear relationship was observed (Fig. 8(b)), indicating that the current density is proportional to *ν*^1/2^. This confirms that the charge transport during the glucose oxidation process is predominantly diffusion controlled.^74^

#### 3.4.4. Optimal oxidation conditions of electrode for glucose detection

The effect of sodium hydroxide (NaOH) concentration on glucose detection was evaluated by amperometric measurements at a glucose concentration of 2 mM. The transition metal–based electrode demonstrated enhanced catalytic oxidation of glucose under alkaline conditions.^73^ As shown in Fig. 8, the anodic peak current increased as the NaOH concentration rose from 0.005 M to 0.1 M. However, further increasing the NaOH concentration caused a decrease in the anodic peak current. Hence, an optimal NaOH concentration of 0.1 M was selected for glucose sensing with the designed electrode.^75^

The effect of pH on the electrochemical behavior of the ZnO/CuO/rGO/GCE for glucose detection was evaluated by cyclic voltammetry (CV) across a pH range of 9–14, as shown in Fig. 10. A 0.1 M NaOH solution served as the supporting electrolyte, and the applied potential was varied from −0.2 V to 1.0 V. The pH of the supporting electrolyte plays a critical role in the electro-oxidation of glucose, and NaOH solutions with pH values of 9, 10, 11, 12, 13, and 14 were carefully prepared for this study.

**Fig. 9:**
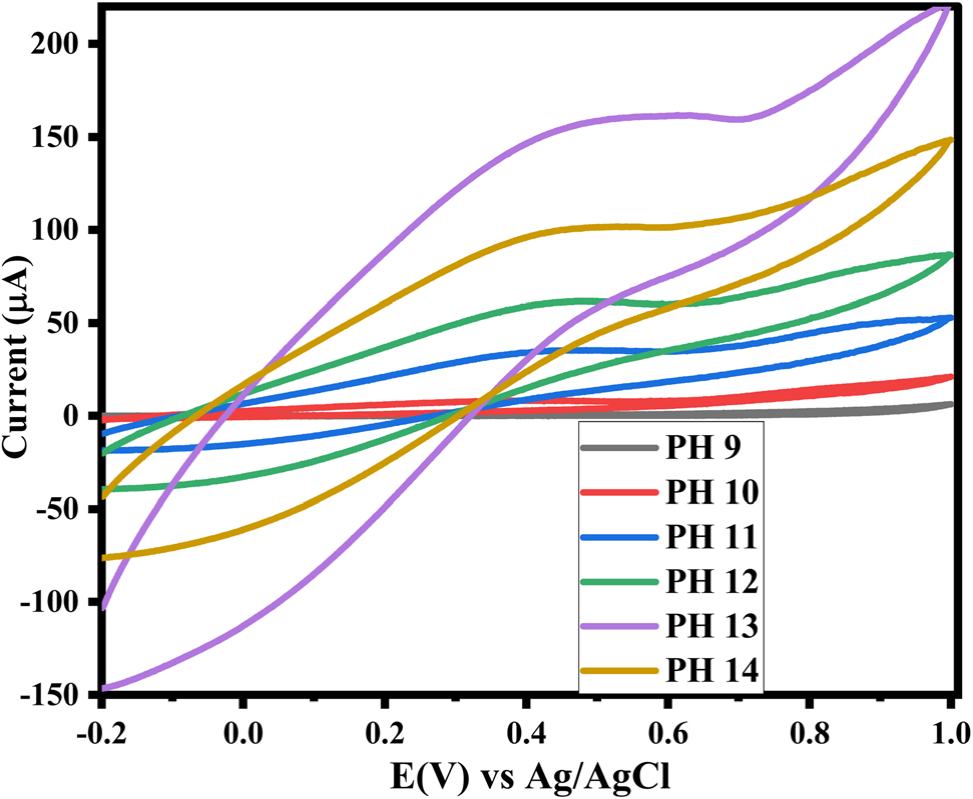
Cyclic voltammograms of ZnO/CuO/rGO/GCE *versus* at different pH (9–14) of NaOH upon addition of 2 mM glucose at scan rate of 50 mV s^−1^.

At pH 9 and 10, no noticeable anodic or cathodic peaks were observed within the applied potential range. At pH 13, the CV response was significantly higher, indicating that this pH provides an ideal environment for the electro-oxidation of glucose due to the favorable availability of OH^−^ ions, which promotes a rapid and efficient reaction. In contrast, at pH 14, the anodic and cathodic peaks decreased due to an excess of OH^−^ ions, which disrupt the electron transfer and hinder the reaction between glucose and the modified ZnO/CuO/rGO/GCE. As illustrated in Fig. 9, this results in a diminished electro-oxidation process. Notably, the anodic and cathodic peak currents at pH 13 were higher than those observed at other pH levels, confirming that pH 13 is the optimum condition for glucose detection with the modified electrode.

**Fig. 10:**
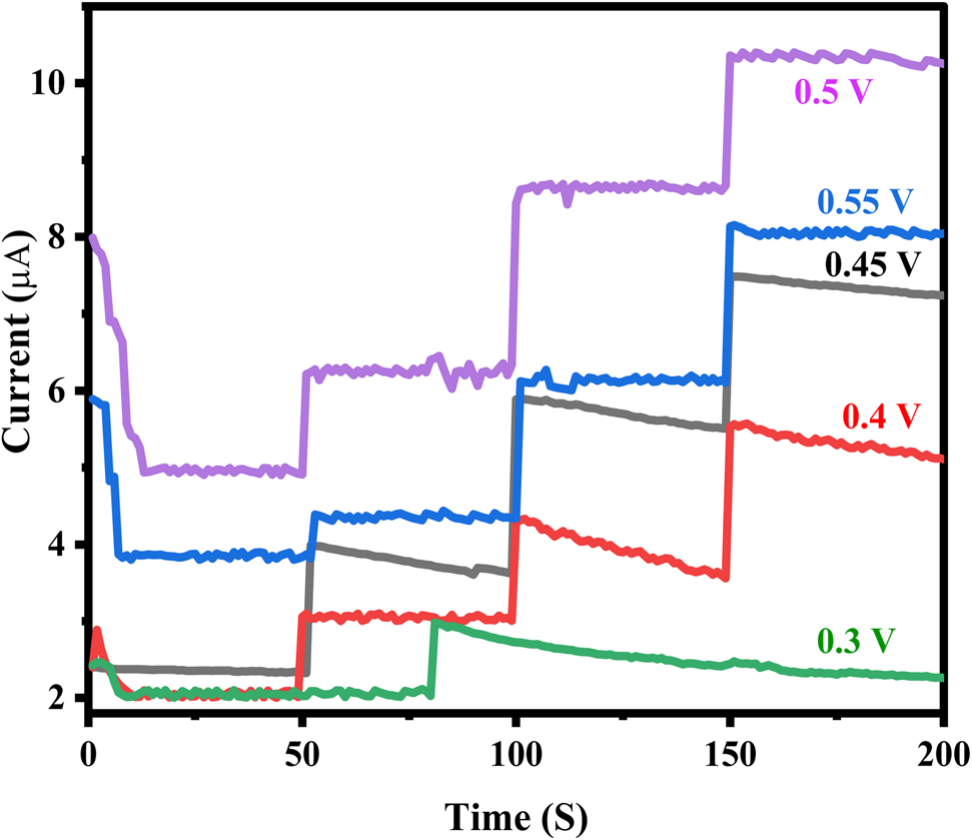
Amperometric (*i*–*t*) graphs to the successive injections of 2 mM glucose at different applied potentials from 0.3–0.55 V.

The amperometric response of the ZnO/CuO/rGO modified GCE was evaluated to determine the optimal potential for glucose detection. The electrode was tested within a potential range of 0.3–0.55 V. As shown in Fig. 10, the anodic current increased sharply between 0.45 V and 0.50 V, indicating a strong glucose response. However, when the potential was increased further to 0.55 V, the amperometric response decreased, suggesting reduced efficiency. The highest sensitivity was observed at an applied potential of 0.50 V, which was selected as the optimal working potential for subsequent experiments. At lower detection potentials, the influence of easily oxidized interfering species was also minimized, further supporting the choice of 0.50 V as the ideal potential for glucose sensing.^76^

#### 3.4.5. Amperometric detection of glucose

The amperometric technique is used for glucose oxidation due to its low noise, high sensitivity, and rapid detection capabilities compared to other methods. In addition, this technique promotes efficient mixing of the sample solution and enhances the transport of analyte to the electrode surface *via* convection, facilitating a faster and more accurate measurement.

**Fig. 11:**
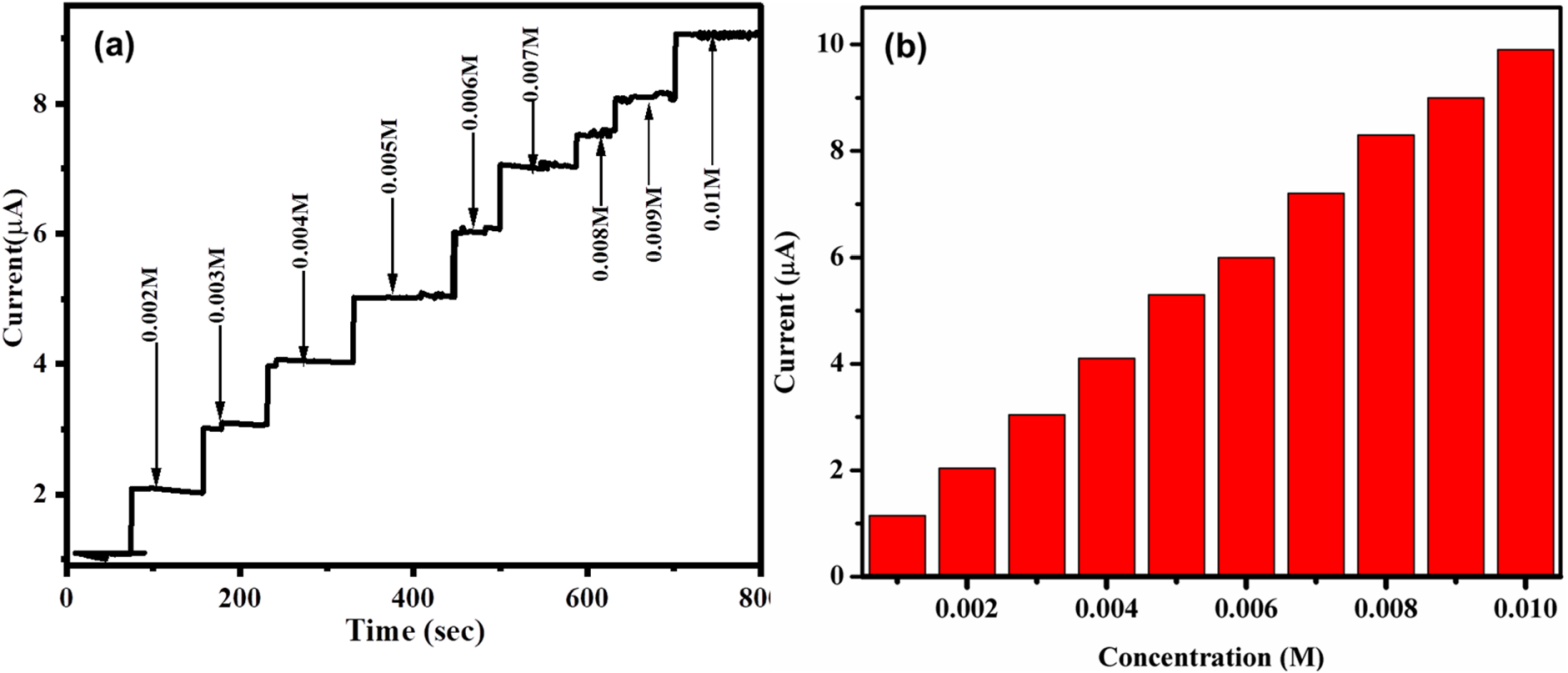
(a) Amperometric *i*–*t* curve of ZnO/CuO/rGO/GCE electrode in response to successive glucose solution additions within a stirred environment of 0.1 M NaOH (pH = 13)at +0.55 V, (b) Linear calibration plot of the corresponding current *versus* glucose concentration.

Fig. 11(a) shows the steady state amperometric response of the ZnO/CuO/rGO/GCE to successive additions of glucose at concentrations ranging from 0.002 M to 0.01 M under continuous stirring in 0.1 M NaOH at an applied potential of +0.5 V. Upon each glucose addition, the modified electrode exhibited a sharp, well defined increase in current, reaching 90% of its steady state value in less than 3 seconds, highlighting its rapid and sensitive detection capabilities. The shape of the current–time steps depends on the glucose concentration in the solution.

The calibration plot of the current response *versus* glucose concentration is presented in Fig. 11(b), demonstrating the sensor's linear behavior over a range of 2–10 mM. The sensitivity was calculated from the slope of the calibration plot normalized to the active surface area of the electrode, yielding a value of approximately 5660 μAM^−1^ cm^−2^. The limit of detection (LOD) of the present sensor was calculated to be 0.54 μM by using the known formula 3*δ*/*s*, where, *δ* – standard deviation of background current of *i*–*t* signal-to-noise ratio of 3S/N = 3; *s*-slope value of the calibration plot. All the low detection limit, sensitivity, and linear range of glucose were contrasted with the available studies as shown in Table 2. As shown in Table 2, the ZnO/CuO/rGO/GCE sensor offers a lower detection limit, higher sensitivity, and a wider linear range compared to previously reported glucose sensors. Its ability to reliably detect glucose in the range of 2–10 mM presents significant advantages for practical applications, including enhanced sensitivity, reduced interference, improved precision, lower cost, and portability making it highly suitable for glucose monitoring in clinical and point of care settings.

**Table 2:** Comparison of the performance of non-enzymatic glucose sensors using a ZnO/CuO/rGO/GCE modified electrode with earlier studies

Electrode materials	Electrode	Fabrication method	Method of detection	Detection potential (V)	Sensitivity (μA mM^−1^ cm^−2^)	LOD (μM)	Linear range (mM)	Ref.
CuO–NiO microfibers	Fluorinetinoxide	Electro spinning	Amp	0.50	3165.3	0.001	3–510	13
CuO–ZnO nanofibers	Pt electrode	Electro spinning and subsequent thermal treatment	Amp	0.50	463.6	0.126	0.8–3880	77
Mesoporous ZnO/NiO	GCE	Thermal annealing	Amp	0.598	120.5	0.5	0.5–6400	78
3D-porousCuO/TiO_2_	Fluorine tin oxide	Electro spinning	Amp	0.70	1321	0.039	10–2000	79
ZnO NRs–CuO	GCE	Hydrothermal	Amp	0.70	1475.5	0.038	0.1–4167	23
NiO/CuO/rGO	GCE	A facile and direct electrochemical method	Amp	0.55	1046	0.5	5–4.85	80
ZnO/Co_3_O_4_/rGO	GCE	Hydrothermal	CV/Am	0.55	1551.38	0.043	0.015–10	44
GO/CuO/nanofibers	Fluorine tin oxide	Hydrothermal	Amp	0.6	1274.8	0.13	0.1–1	81
GR–CuO NPs	GCE		Amp	0.6	1065	1	1 μM–8 mM	35
ZnO/CuO/rGO/GCE	GCE	Hydrothermal	Amp	0.5	5660	0.54	2–10	This work

#### 3.4.6. Interference analysis

**Fig. 12:**
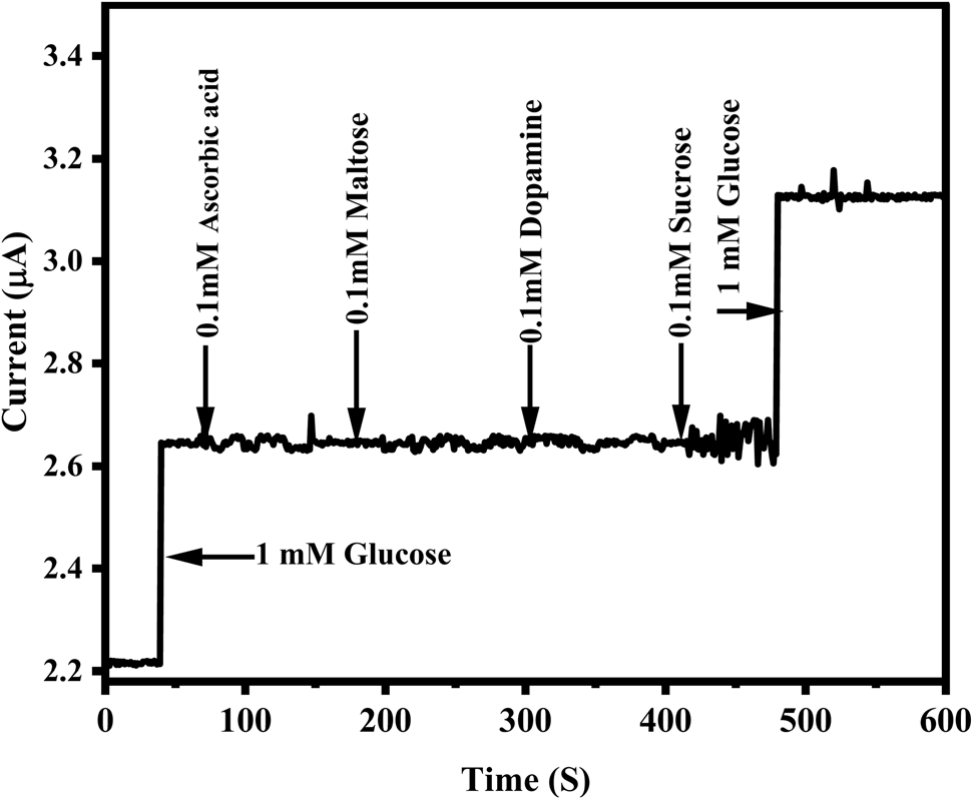
Shows the amperometric response of the ZnO/CuO/rGO/GCE in 0.1 M NaOH (pH 13) when glucose (1 mM), uric acid (0.1 mM), ascorbic acid (0.1 mM), maltose (0.1 mM), sucrose (0.1 mM), dopamine (0.1 mM), and 1 mM glucose are successively added.

The anti-interference ability of a sensor is critical for its practical application. A major challenge for non-enzymatic glucose sensors is the potential interference caused by other organic compounds in physiological fluids, which can oxidize at similar potentials. Common interfering species such as uric acid, ascorbic acid, sucrose, maltose, and dopamine can affect the direct electrochemical detection of glucose, especially at an applied potential of 0.55 V. To assess selectivity, amperometric measurements were performed in the presence of 1 mM glucose and 0.1 mM of each interfering species in 0.1 M NaOH (pH 13). As shown in Fig. 12, the sensor exhibited a significant current response upon the addition of glucose, while the responses to uric acid, ascorbic acid, dopamine, maltose, and sucrose were negligible. The results clearly indicate excellent selectivity for glucose, as no noticeable current changes were observed for the common interferents. The slight current variations observed for maltose and sucrose can be attributed to competitive interactions at the sensor surface, as well as the sensor's higher specificity towards glucose due to its molecular structure and stronger interaction with the sensor's active sites. Overall, the results confirm that the presence of common interfering species does not affect the sensor's performance, highlighting its strong anti-interference characteristics and making it highly suitable for accurate glucose detection in complex biological samples.^82^

#### 3.4.7. Stability, reproducibility, and repeatability of electrodes

The stability of the ZnO/CuO/rGO modified electrodes was evaluated by monitoring their current response to 2.0 mM glucose over a 10-day period. The electrodes were stored under ambient air conditions, and the current was measured every two days. The sensors demonstrated excellent long term stability, with only a 10.5% decrease in the current response after 10 days.

Reproducibility was assessed by recording the current response to 2 mM glucose across multiple trials. The relative standard deviation (RSD) of six consecutive measurements with the same ZnO/CuO/rGO modified GCE was 2.9%, indicating strong intra electrode reproducibility. In an inter electrode reproducibility test, five identically prepared electrodes were analyzed, yielding an RSD of 4.2%. Additionally, intra electrode reproducibility was further confirmed with an RSD of 3.8% (*n* = 5) for 2 mM glucose. These results clearly demonstrate the long term stability, reproducibility, and reliability of the ZnO/CuO/rGO modified electrodes for glucose sensing.

#### 3.4.8. Application in real sample analysis

**Fig. 13:**
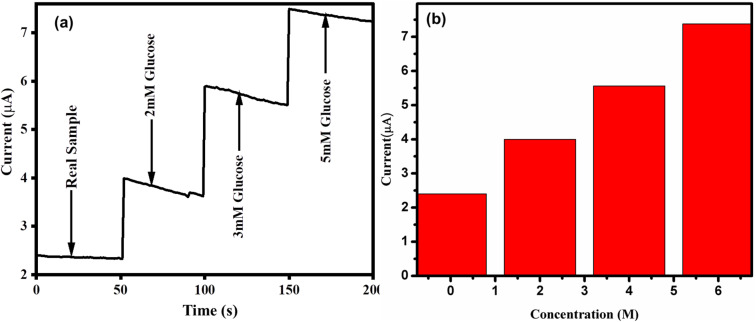
(a) Amperometric *i*–*t* response before and after addition of glucose to blood samples at an applied potential of 0.5 V (b) calibration curve plot of the obtained current *versus* serum sample.

The ZnO/CuO/rGO modified electrode was used for the real time analysis of glucose in human blood samples, as shown in Fig. 13. The unspiked glucose concentration in diabetic blood samples was determined to be 125 mg dL^−1^ (6.937 mM) based on three replicate measurements using a commercially available spectrophotometer. For the analysis, 0.5 mL of blood serum was diluted with 0.1 M NaOH solution to a total volume of 10 mL. Recovery studies were conducted by spiking the diluted blood samples with standard glucose solutions of 2, 3, and 5 mM, within the linear range of measurement. As presented in Table 3, the glucose concentrations measured by the modified electrode closely matched those obtained from the spectrophotometric method used in clinical diagnostics, with a relative difference of only 1.7%. The analytical recovery ranged from 100.55% to 102.65%, indicating high precision and accuracy. These values fall well within the acceptable range of 80–120% stipulated by the WHO guidelines for analytical method validation. These results confirm that the proposed sensor is highly precise, accurate, and reliable for the detection of glucose in real human blood samples, making it a promising candidate for clinical and point of care applications.

**Table 3:** Conditions for operation and amperometric analysis of glucose levels added to human blood samples (*n* = 3)

Blood sample by spectrophotometer (mM)	Spiked concentration of glucose (mM)	Determined glucose by ZnO/CuO/rGO(mM)	RSD (%)	Recovery (%)
6.937	0	6.92	3.85	—
	2	8.99	3.35	102.65
	4	10.99	2.62	101.32
	6	12.97	2.87	100.55

## 4. Conclusion

A simple hydrothermal method was used to synthesize the ZnO/CuO/rGO nanohybrid, and its optical properties, vibrational modes, and structural characteristics were confirmed by XRD, FTIR, and UV-vis spectroscopy. The resulting nanohybrid offered a large exposed surface area, high electrical conductivity, and rapid electron transport, making it highly effective for non-enzymatic glucose detection. The sensor achieved a high sensitivity of 5660 μA M^−1^ cm^−2^ and a low detection limit of 0.54 μM. In addition, the ZnO/CuO/rGO/GCE demonstrated remarkable catalytic activity toward glucose oxidation, combined with excellent stability, selectivity, sensitivity, and reproducibility outperforming many previously reported sensors. Its performance in real blood samples was highly accurate, aligning closely with spectrophotometric results used in clinical settings, highlighting its potential for practical glucose monitoring applications.

## Ethical statement

All experiments were performed in accordance with the guideline of national research ethics review guideline fifth edition: Federal democratic republic of Ethiopia and experiments were approved by the ethics committee at Mattu University. Informed consents were obtained from human participants of this study.

## Author contributions

G. S. W conceived the research. K. A. W., S. A., G. S. W. and N. M. D. conducted the experiments. G. S. W., S. A., N. M. D., B. A. H., A. M. D and T. W. M. analyzed the results. G. S. W., S. A., N. M. D., and B. A. H. prepared the manuscript. All authors reviewed the manuscript.

## Conflicts of interest

The authors declare no competing financial interests. We confirm that the manuscript has been read and approved by all named authors. We confirm that the order of authors listed in the manuscript has been approved by all named authors.

## Data Availability

All data generated in this study are available in the article. Furthermore, additional and original files are available from the authors upon reasonable request.
